# Recombination and peak jumping

**DOI:** 10.1371/journal.pone.0193123

**Published:** 2018-03-01

**Authors:** Kristina Crona

**Affiliations:** American University, Washington DC, United States of America; Universitat Pompeu Fabra, SPAIN

## Abstract

We show that genetic recombination can be a powerful mechanism for escaping suboptimal peaks. Recent studies of empirical fitness landscapes reveal complex gene interactions and multiple peaks. However, classical work on recombination largely ignores the effect of complex gene interactions. Briefly, we restrict to fitness landscapes where the global peak is difficult to access. If the optimal genotype can be generated by shuffling genes present in the population, then recombination will produce the genotype. If, in addition, recombination is sufficiently rare, then the proportion of the genotype is expected to increase. Specifically, we consider landscapes where shuffling of suboptimal peak genotypes can produce the global peak genotype. The advantage of recombination we identify has no correspondence for 2-locus systems or for smooth landscapes. The effect of recombination indicated is sometimes extreme, also for rare recombination, in the sense that shutting off recombination could result in the organism failing to adapt. A standard question about recombination is whether the mechanism tends to accelerate or decelerate adaptation. However, we argue that extreme effects may be more important than how the majority falls. In a limited sense, our result can be considered a support for Sewall Wright’s view that adaptation sometimes works better in subdivided populations.

## Introduction

Recent studies of empirical fitness landscapes reveal complex gene interactions and multiple peaks, e.g. [1–4]. However, most studies of recombination restrict to 2-locus systems or smooth fitness landscapes.

We are interested in the effects of recombination especially for complex fitness landscapes. Recombination may accelerate or decelerate adaptation, depending on properties of the fitness landscapes and population size. Recombination has no impact in the absence of linkage disequilibrium, i.e., non-random associations, e.g. [5].

However, epistasis (gene interactions) may cause linkage disequilibrium. It is intuitively clear that negative epistasis for two beneficial mutations may imply that recombination speeds up adaptation. Briefly, the negative epistasis means that the double mutant combining the beneficial mutations has lower fitness than independent fitness effects would imply. Consequently, the double mutant will be underrepresented during the course of adaptation. Because of linkage disequilibrium, i.e., the relatively low frequency of double mutants, recombination will tend to generate double mutants rather than splitting them up. The result may be faster adaptation.

Another potential advantage of recombination is the Fisher-Muller effect. In the absence of recombination, beneficial mutations may vanish from the population because of clonal interference. For instance, if two single mutants of high fitness co-exist in a population, then one of them may outcompete the other. However, recombination can incorporate beneficial mutations in the same genome, thereby preventing loss of genetic variation due to clonal interference. The Fisher-Muller effect is important only under some conditions, and the topic is quite complex, e.g. [5].

Several other potential advantages of recombination have been discussed in recent work on complex fitness landscapes, including enhanced capacity for valley crossing in the case where deleterious mutations combine to a global peak [6], transitory advantages of recombination and the Red queen hypothesis [7], and the Weismann effect [7, 8], i.e, the effect of increased offspring variation. Most of the potential advantages considered are present already for 2-locus systems or smooth fitness landscapes. However, there is no reason to believe that evolutionary dynamics for multi-locus systems with complex gene interactions can be reduced to lower dimensional dynamics.

Interestingly, empirical studies discuss strictly higher dimensional effects of recombination for complex fitness landscapes [9]. The author suggests that HIV may have arisen, in part, through past recombination between primate lentiviruses, discussing the spread of chimeras between Simian immunodeficiency viruses (SIV) for various primates. The spread of recombinant viruses, regardless of whether within or between species, may represent evolutionary jumps from suboptimal peaks to genotypes of higher fitness.

By peak jumping we understand that the global peak genotype can be generated by shuffling of suboptimal peak genotypes. The possibility of peak jumping and some closely related scenarios were briefly discussed in [10, 11], see also [12]. A very special case of peak jumping has also been studied in the contexts of modules [13]. The advantage of recombination the authors found depends on physical linkage. However, the evolutionary role of peak jumping in general has received limited attention.

Here we develop a theoretical framework for peak jumping as a mechanism. We explore the magnitude of the effect in simulations and provide some empirical support. Our study is intended to clarify when peak jumping could be important, as well as to provide guidance for further empirical research.

## Theoretical results

Throughout the paper, we will consider haploid biallelic *L*-locus populations. Let Σ = {0, 1} and let Σ^*L*^ denote bit strings of length *L*. Σ^*L*^ represents the genotype space. In particular,

$$\begin{array}{c} \Sigma^{2}=\left\{00,10,01,11\right\}\;\text{and}\;\Sigma^{3}=\left\{000,100,010,001,110,101,011,111\right\}. \end{array}$$

We define a fitness landscape as a function $w:\Sigma^{L}\mapsto R$, which assigns a fitness value to each genotype [14]. The fitness of the genotype *g* is denoted *w*_*g*_. Roughly, *w*_*g*_ measures the expected contribution to the next generation for individuals of genotype *g*. The wild-type (or ancestral genotype) is assumed to have fitness 1, and fitness is multiplicative in the absence of gene interactions.

The metric we consider is the Hamming distance, meaning that the distance between two genotypes equals the number of positions where the genotypes differ. In particular, two genotypes are adjacent, or mutational neighbors, if they differ at exactly one position. We will use fitness graphs (see [15] for an extensive discussion) as a representation of coarse aspects of fitness landscapes. Roughly, the nodes represent genotypes, and each arrow points toward the more fit genotype. A fitness landscape is smooth if it can be represented by a fitness graph where all arrows point up, or equivalently, if any shortest walk to the peak is an uphill walk [16, 17].

We are interested in fitness landscapes where peak jumping has a critical role. Intuitively, there are obstacles for adaptation in such landscapes, but shuffling of genotypes can resolve the problem. For simplicity, we assume that the global peak can be produced by shuffling of suboptimal peaks. Importantly, we do not assume any physical linkage between loci. The motivation is that we want to investigate the potential advantage of recombination under the most difficult conditions possible, so as to obtain an *underestimate* of the advantage. Indeed, as pointed out in [13], physical linkage is likely to increase the probability that beneficial “blocks” are incorporated into the same genome through recombination. However, tight linkage cannot always be assumed, and we aim for a general understanding of peak jumping.

More formally, consider an adapting organism. After a recent change in the environment, the wild-type, denoted **0** = 0 … 0, no longer has maximal fitness. We assume that there is no sequence of beneficial single mutations starting at the wild-type and ending at the global peak. Equivalently, there is no path to the global maximum in the fitness graph. Some authors refer to this property as the global peak not being evolutionary accessible.

At the same time, it should be possible for a Darwinian process to produce the global peak genotype by a combination of beneficial mutations and gene shuffling. Specifically, we assume that two (or more) suboptimal peaks are evolutionary accessible, and that the global peak can be generated by shuffling of the suboptimal peaks.

The general conditions on the fitness landscape are summarized below, followed by brief interpretations in biological terms, and then several explicit examples of landscapes for an intuitive understanding.

One should keep in mind that population parameters are important for the evolutionary outcome as well, (see Population Conditions and related discussions later in this section).

**Main assumptions.** Let *g*_max_ denote the genotype of maximal fitness in Σ^*L*^. There exist genotypes *g*^1^, …, *g*^*L*^ ∈ Σ^*L*^ such that:

(L1) There is no path from **0** to the *g*_max_ in the fitness graph.
(L2)
  $$\begin{array}{c} g_{\text{max}}=g_{1}^{1}\cdots g_{L}^{L}, \end{array}$$
  where $g_{i}^{k}$ is the *i*th bit of *g*^*k*^ (notice that the elements *g*^*k*^ are not necessarily different).
(L3) For each *k*, there exists a path in the fitness graph from **0** to *g*^*k*^.
(L4) Each *g*^*k*^ is a suboptimal peak.
(L5) *w*_*g*_ differs from zero for intermediate genotypes.

In brief, L1 means that an adapting population could be trapped at a suboptimal peak, L2 that the global peak *g*_max_ can be obtained by a sequence of recombination events using genotypes in {*g*^*k*^}. Conditions L3 and L4 imply that the genotypes *g*^*k*^ have a reasonable chance to encounter each other for large populations (this aspect will be discussed later). Finally, L5 is more technical. The condition makes sure that the evolutionary process can take advantage of intermediates for reaching the global peak.

Landscapes satisfying L1-L5 are of interest because of the potential advantage of recombination. (Note that the conditions L1-L5 are neither necessary, nor sufficient, for recombination to be advantageous because of peak jumping. This topic will be discussed in more detail.)

Our first observation is that no landscape satisfies L1-L5 in the 2-locus case. Indeed, if L1 holds then genotype 11 is a global peak. In addition, the single mutants 10, 01 are deleterious. In summary

$$\begin{array}{c} w_{10},w_{01}<w_{00}\;\text{and}\;w_{10},w_{01}<w_{11}. \end{array}$$

**Fig 1:**
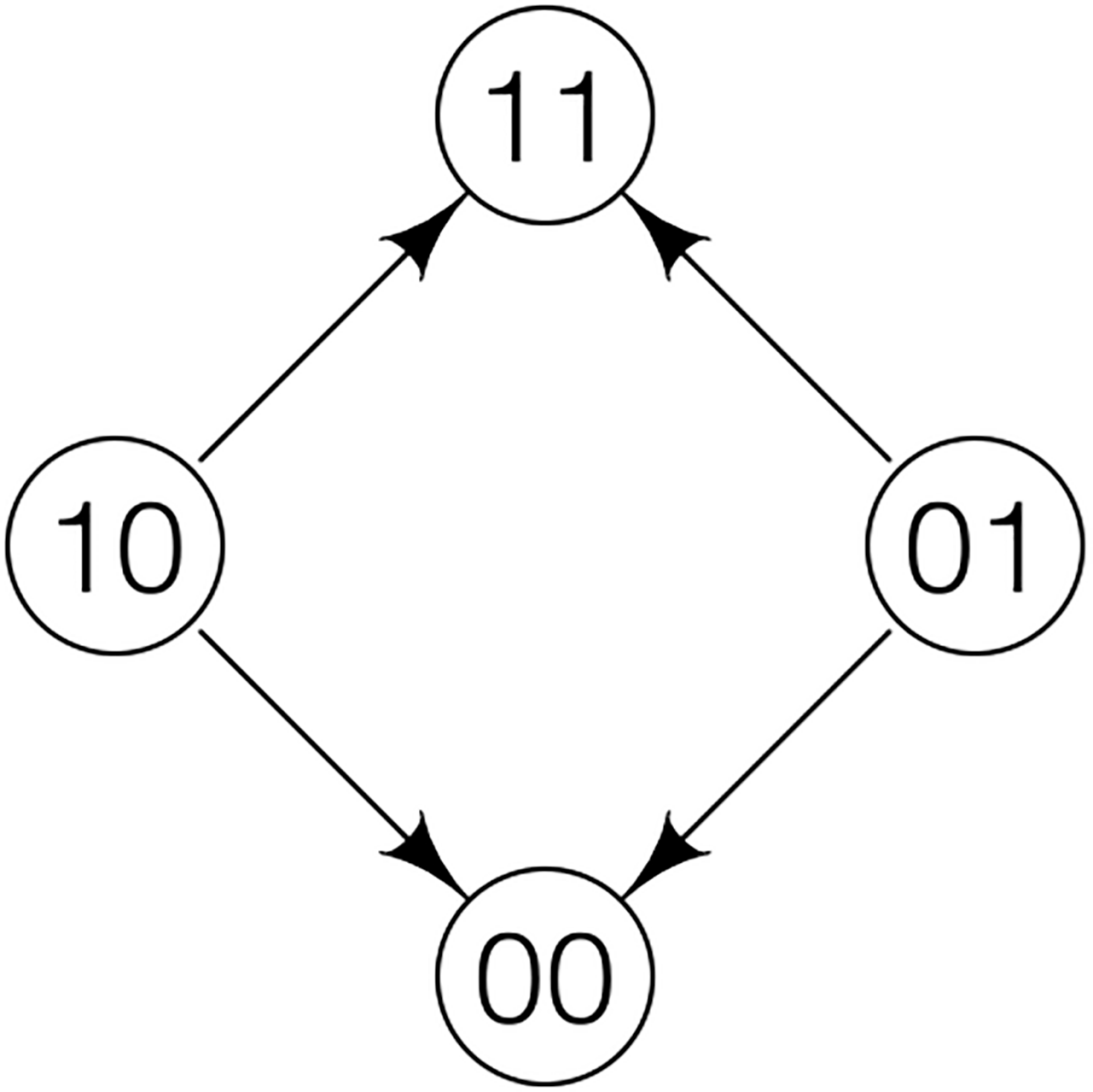
The sole fitness graph for 2 loci satisfying condition L1.

**Fig 2:**
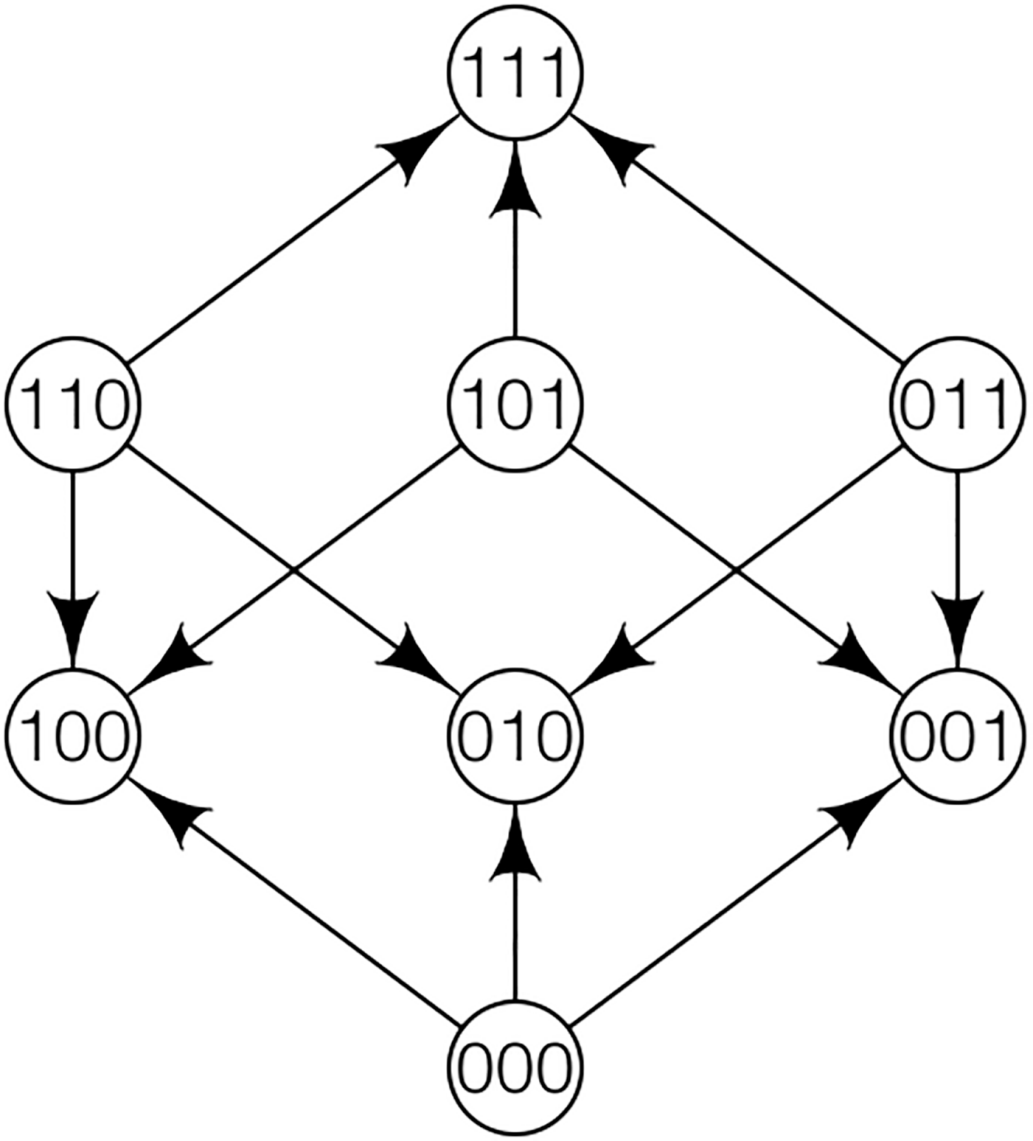
A fitness graph for 3 loci. The corresponding fitness landscapes satisfy L1-L5.

**Fig 3:**
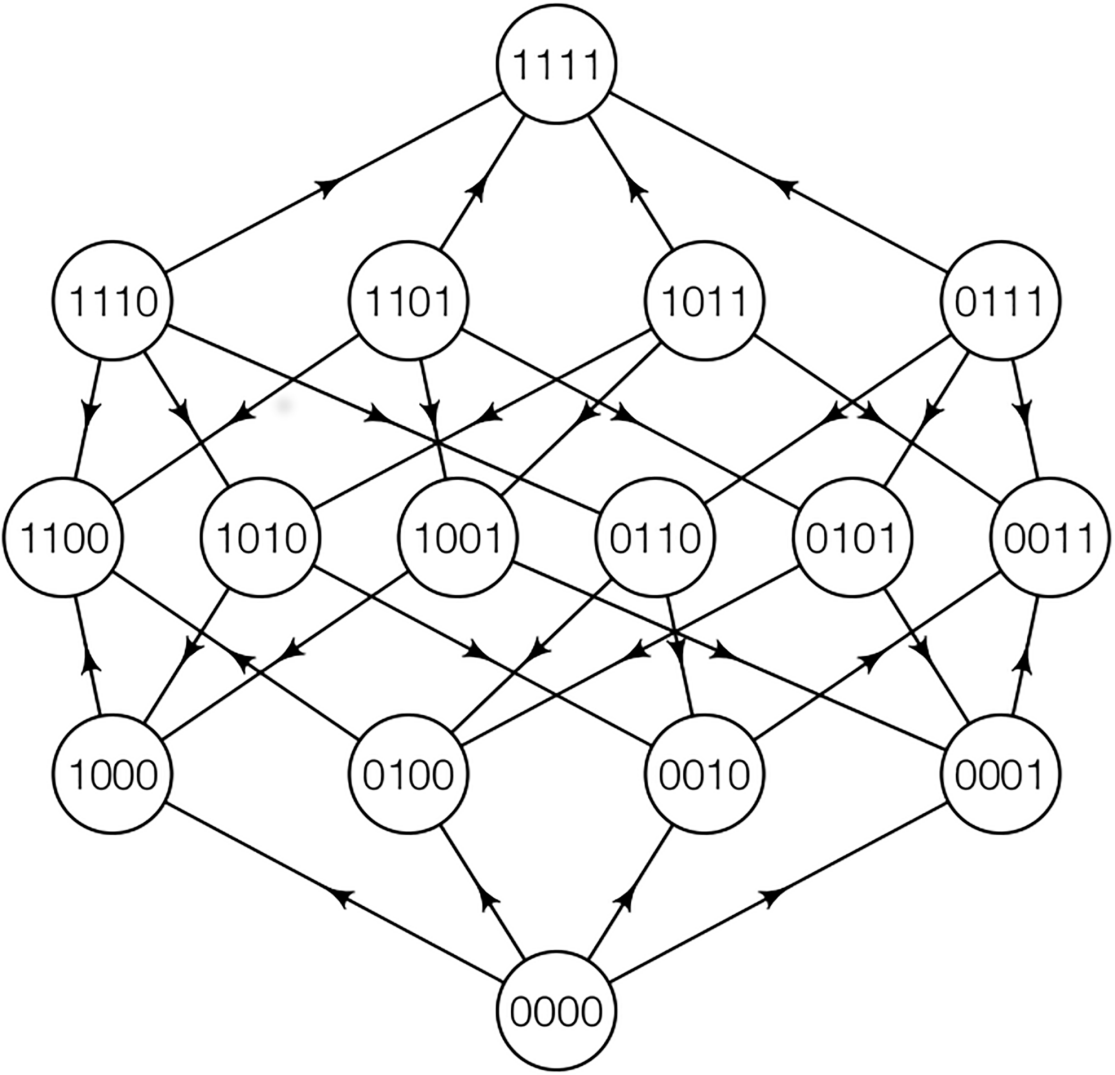
A fitness graph for 4 loci. The corresponding fitness landscapes satisfy L1-L5.

Fig 1 shows the fitness graph determined by these conditions. Clearly not all of the conditions L1-L5 are satisfied. However, L1-L5 are satisfied for Examples 1 and 2 (see Figs 2 and 3).

**Example 1.** *For L* = 3 *consider a case where 111 is the global peak and 100, 010, 001 are suboptimal peaks. Specifically, the genotypes 100, 010, 001 have higher fitness than the wild-type 000. The double mutants 110, 101, 011 have lower fitness than the wild-type*.

**Example 2.** *For L* = 4, *consider a case where 1100 and 0011 are suboptimal peaks and 1111 the global peak*.

*Specifically, the genotypes 1000, 0100, 0010, 0001 have higher fitness than the wild-type 0000. The double mutants 1100, 0011 have higher fitness than the single mutants. The remaining double mutants 1010, 0101, and the triple mutants 1110, 1101, 1011, 0111 have low fitness*. *In summary*,

$$\begin{array}{c} w_{1000},w_{0100},w_{0010},w_{0001}>w_{0000}\\ w_{1100}>w_{1000},w_{0100},\;w_{0011}>w_{0010},w_{0001},\\ w_{1111}>w_{1100},w_{0011},\\ w_{1001},w_{1010},w_{0110},w_{0101},w_{1110},w_{1110}w_{1101}w_{1011}w_{0111}>w_{0000}. \end{array}$$

The double peaked 2-locus case (Fig 1) and Example 2 (Fig 3) have some similarities. Indeed, in both cases there are obstacles for adaptation from the wild-type to the global maximum. In the 2-locus case, the frequencies of 10 and 01 are expected to be very low, so recombination is not a powerful generator of the optimal genotype 11. In contrast, in Example 2 both 1100 and 0011 are peak genotypes, and recombination may be a powerful generator of the optimal genotype 1111. Informally, one needs to combine “rare and rare” in the 2-locus case, and “abundant and abundant” in Example 2.

The potential advantage of recombination for an Example 2 population should not be underestimated. For instance, consider a relatively small subdivided population. Then each subpopulation would be likely to end up at 1100 or 0011. Recombination could generate 1111, as soon as there is some migration between the subpopulations. However, in absence of recombination, the expected time before 1111 appears would be very long. Indeed, a double mutation or some other rare scenario would be necessary. A relatively small population may fail to produce 1111 genotypes altogether.

One can ask how prevalent fitness landscapes of the type described in Example 2 are. The TEM-family of *β*-lactamases provides an interesting example. TEM-1 is the wild-type in the system, and approximately 200 mutants have been found clinically, for a record see http://www.lahey.org/Studies/temtable.asp.

Consider TEM-1, the 4-tuple mutant TEM-50 and intermediates. The clinically found subset of these 16 genotypes are compatible with the Example 2 fitness graph. More precisely, the clinically found alleles can be represented as:

$$\begin{array}{c} 0000,1000,0100,0010,0001,1100,0011,1111. \end{array}$$

In particular, none of the triple mutants have been found clinically. It seems reasonable to interpret the absence of mutants as an indication of low fitness in a natural setting. If that interpretation is correct, then the set of clinically found mutants is compatible with the Example 2 fitness graph.

Recombination will generate the optimal genotype for Example 2 landscapes in many cases. Other fitness landscapes satisfying conditions L1-L5 may not be quite as favorable. However, the basic mechanism is similar.

For instance, consider Example 1. From the three peak genotypes 100, 010, 001, recombination could produce some of the intermediates 110, 101, 011, and in the next step 111. For instance, 100 and 010 could produce 110, and then 110 and 001 could produce 111. Notice that this scenario requires that *w*_110_ > 0. In addition, the population structure would have to allow for different genotypes to recombine.

In summary, recombination has the potential to generate the optimal genotypes for L1-L5 landscapes. As we have seen, some restrictions on population structure and size are usually necessary. Notice also that non-recombining populations will get trapped only if genetic diversity is somewhat restricted so that double mutations are rare. It is therefore reasonable to focus only on L1-L5 landscapes which also satisfy the following population conditions (recall that the elements *g*^*k*^ are suboptimal peaks, that are also evolutionary accessible).

**Population Conditions**.

(P1) The potential genetic diversity is not extreme (double mutations should still be rare).
(P2) The *g*^*k*^ elements are likely to co-exist, encounter each other and recombine during some stage of adaptation. (Strictly speaking, we need to assume that also the resulting intermediates are likely to recombine with the *g*^*k*^ elements, and likewise for subsequently produced intermediates. However, this condition follows automatically from the previously stated conditions in most cases of interest.)

The condition P2 imposes requirements on population structure as well as fitness landscape. For Examples 1 and 2, the requirement on the population structure is modest. In general, the assumption that *g*^*k*^ are evolutionary accessible peaks makes it plausible that they will be present in the population for an extended period of time (provided the population is sufficiently diverse). Nevertheless, it is obviously difficult to determine exactly when P2 holds. For instance, fine-scaled properties such as migration rates for subdivided populations will determine whether or not P2 holds. Peak jumping is important for many landscapes satisfying L1-L5, but one needs to determine the requirements on the population parameters from case to case.

We have so far discussed how recombination can generate optimal genotypes. However, even if the optimal genotype appears in a population, it is far from obvious that the genotype will go to fixation. If optimal genotypes recombine poorly, they may stay rare in the population.

We define the recombination rate 0 ≤ *r* ≤ 1 as the proportion of individuals that recombine. In particular, *r* = 0 refers to a non-recombining population and *r* = 1 to a population with full recombination. Recall that no physical linkage is assumed (see the Methods section for more details).

**Main result.** Consider populations which satisfy conditions L1–L5, P1 and P2. Then recombination will speed up adaptation provided that the recombination rate *r* > 0 is sufficiently small.

The argument is fairly straight forward. In the absence of recombination a population will be trapped at a suboptimal peak for a very long time. Rare events, such as double mutations, will be necessary for escapes. In contrast, recombination will generate the optimal genotype within a relatively short time interval under our assumptions. As soon as the optimal genotype appears, Theorem 1 [below] shows that the proportion is expected to grow provided that *r* is sufficiently small. (A result closely related to Theorem 1 can also be found in [18].)

**Theorem 1**. *For a fitness landscape* $w:\Sigma^{L}\mapsto R$, *let g*_max_ *be the genotype of maximal fitness w*_max_, *and let w*_max′_ *denote the second to highest fitness. Consider a population with genotypes in* Σ^*L*^ *and recombination rate r. If g*_max_ *is present in a population, then its proportion is expected to increase provided that*

$$\begin{array}{c} r<\frac{w_{\max}-w_{\max^{\prime }}}{w_{\max}+w_{\max^{\prime }}}. \end{array}$$

The proof of Theorem 1 depends on the following lemma.

**Lemma 1.** *For a fitness landscape* $w:\Sigma^{L}\mapsto R$, *let g*_max_ *be the genotype of maximal fitness and let r denote the recombination rate. Then the number of individuals with genotypes in the set* Σ^*L*^ \ {*g*_max_} *will not increase more than by a factor of* (1 + *r*) *as a result of recombination*.

*Proof*. The argument depends on considering how recombination can generate genotypes in Σ^*L*^ \ {*g*_max_} i.e., genotypes different from *g*_max_.

The recombining pairs are of three types:

$$\begin{array}{c} \left\{g_{\max},g_{\max}\right\},\;\left\{g_{\max},g:g\neq g_{\max}\right\},\;\left\{g,g^{\prime }:g,g^{\prime }\neq g_{\max}\right\}. \end{array}$$

For a pair of the first type, recombination has no effect since both individuals complex have the same genotype. For the third type, the number of individuals with genotypes different from *g*_max_ will not increase. For the second type, recombination will result in at most 2 individuals with genotypes different from *g*_max_. In summary, only a pair of the second type is associated with a potential increase of individuals with genotypes in Σ^*L*^ \ {*g*_max_}, and the maximal net effect is one more individual. The number of individuals with genotypes different from *g*_max_ cannot increase more than in the case where all recombining pairs are of the second type, i.e., more than by a factor of (1 + *r*).

*Proof of Theorem 1*.

*Proof*. For simplicity, we consider discrete generations. The proportions *p*_1_ of *g*_max_ genotypes, and *p*_2_ of remaining genotypes in a new generation can be expressed as

$$\begin{array}{c} p_{1}=\frac{C_{1}\tilde{p_{1}}}{C_{1}\tilde{p_{1}}+C_{2}\tilde{p_{2}}},\;p_{2}=\frac{C_{2}\tilde{p_{2}}}{C_{1}\tilde{p_{1}}+C_{2}\tilde{p_{2}}}, \end{array}$$

where $\tilde{p_{1}},\tilde{p_{2}}$ are the proportions in the previous generation. For estimating *C*_1_ and *C*_2_, one needs to analyze the effect of selection and recombination. (For simplicity we ignore other factors, since they usually have very small effects compared to selection and recombination.) From considering *g*_max_ individuals that do not recombine, one concludes that

$$C_{1}\geq w_{\max}\left(1-r\right).$$

The upper bound of *C*_2_ depends on *r* and the maximal fitness *w*_max′_ of elements in Σ^*L*^ \ {*g*_max_}. By assumption,

$$C_{2}\leq w_{\max^{\prime }}\;\left(1+r\right)$$

by Lemma 1. It follows that the *g*_max_ proportion increases if

$$w_{\max}\;\left(1-r\right)\;>\;w_{\max^{\prime }}\;\left(1+r\right)$$

or if

$$\begin{array}{c} r<\frac{w_{\max}-w_{max^{\prime }}}{w_{\max}+w_{\max^{\prime }}}. \end{array}$$

## Simulations

According to the main result, recombination has the potential to speed up adaptation because of peak jumping. However, it is necessary that recombination is rare enough and that the population is sufficiently diverse. In particular, according to Theorem 1, the proportion of the optimal genotype in the population is expected to increase provided that the recombination rate *r* is small enough. We performed simulations for more quantitative precision.

The starting point for most of our simulations is a homogeneous population. We use a Fisher-Wright type model to simulate evolution (a forward simulation) and estimate the probability that the global peak genotype reaches fixation. We simulated several fitness landscapes and population structures. The landscapes have some features in common. All of them have 2 or 3 suboptimal peaks, and the optimal genotype can be produced by shuffling the suboptimal peak genotypes. Fig 3 shows a fitness graph for landscapes with 2 suboptimal peaks, and Fig 2 for 3 suboptimal peaks.

The simulations focus mainly on subdivided populations. This is justified since many microbial populations are subdivided, with some flux between subpopulations. For instance, whenever water or other fluids coalesce, microbes will mix as well. Subpopulations tend to be relatively small and may therefore get trapped at suboptimal peaks for extended periods of time. Mixture of suboptimal peak populations is consequently a recurrent phenomenon for many subdivided populations.

**Fig 4:**
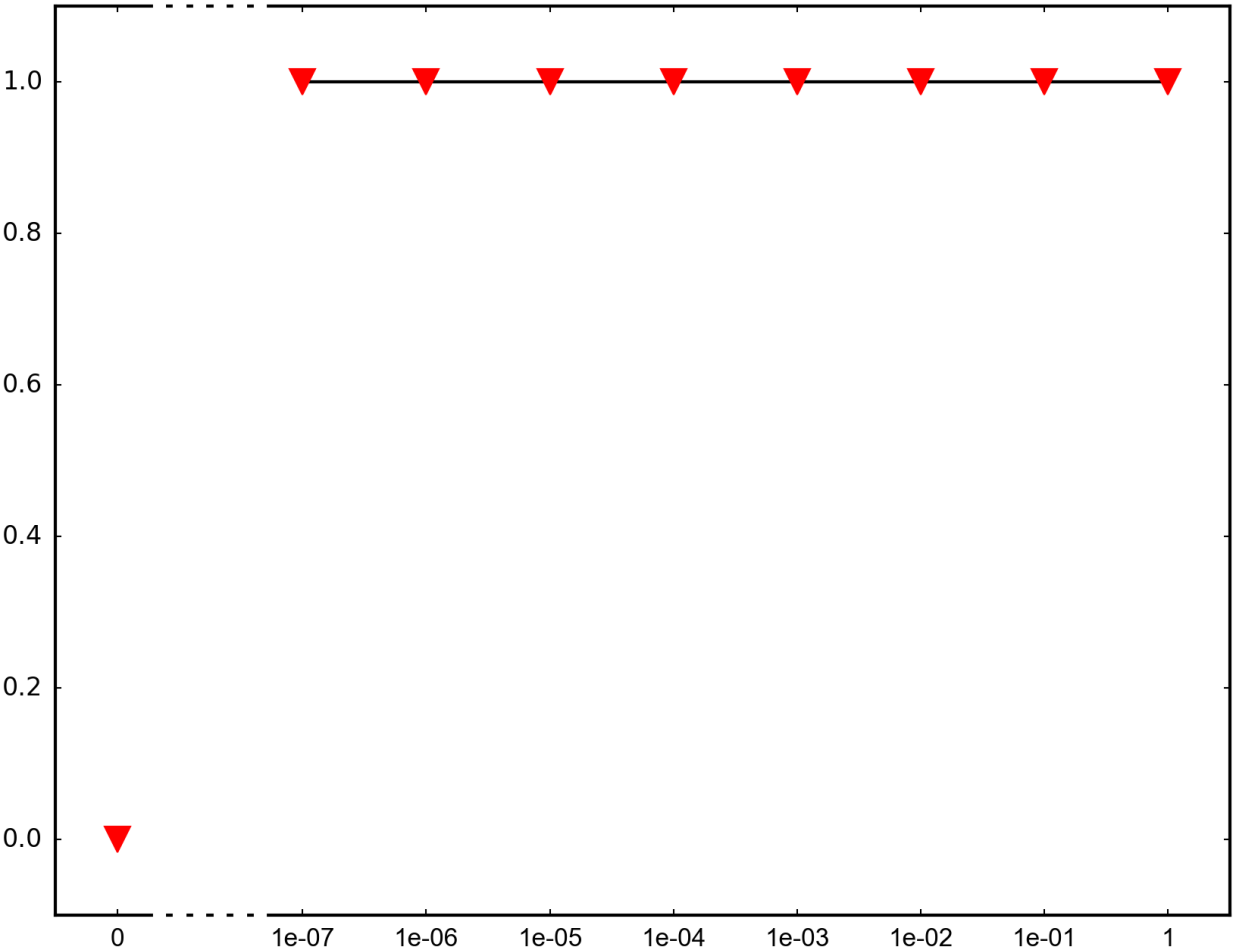
The horizontal axis shows the recombination rate *r* and the vertical axis the probability that the global peak genotype reaches fixation (and likewise for Figs 5–12). The graph shows the effect of recombination for a 4-locus system with 2 suboptimal peaks. The genotype 1111 is the global peak, and the genotypes 1100 and 0011 are suboptimal peaks. Initially, the population is equally distributed on the two suboptimal peaks. The simulation is intended to capture a critical part of an evolutionary scenario for subdivided populations with migration. Specifically, two genetically different subpopulations mix. See the Methods section for more details on the simulations.

**Fig 5:**
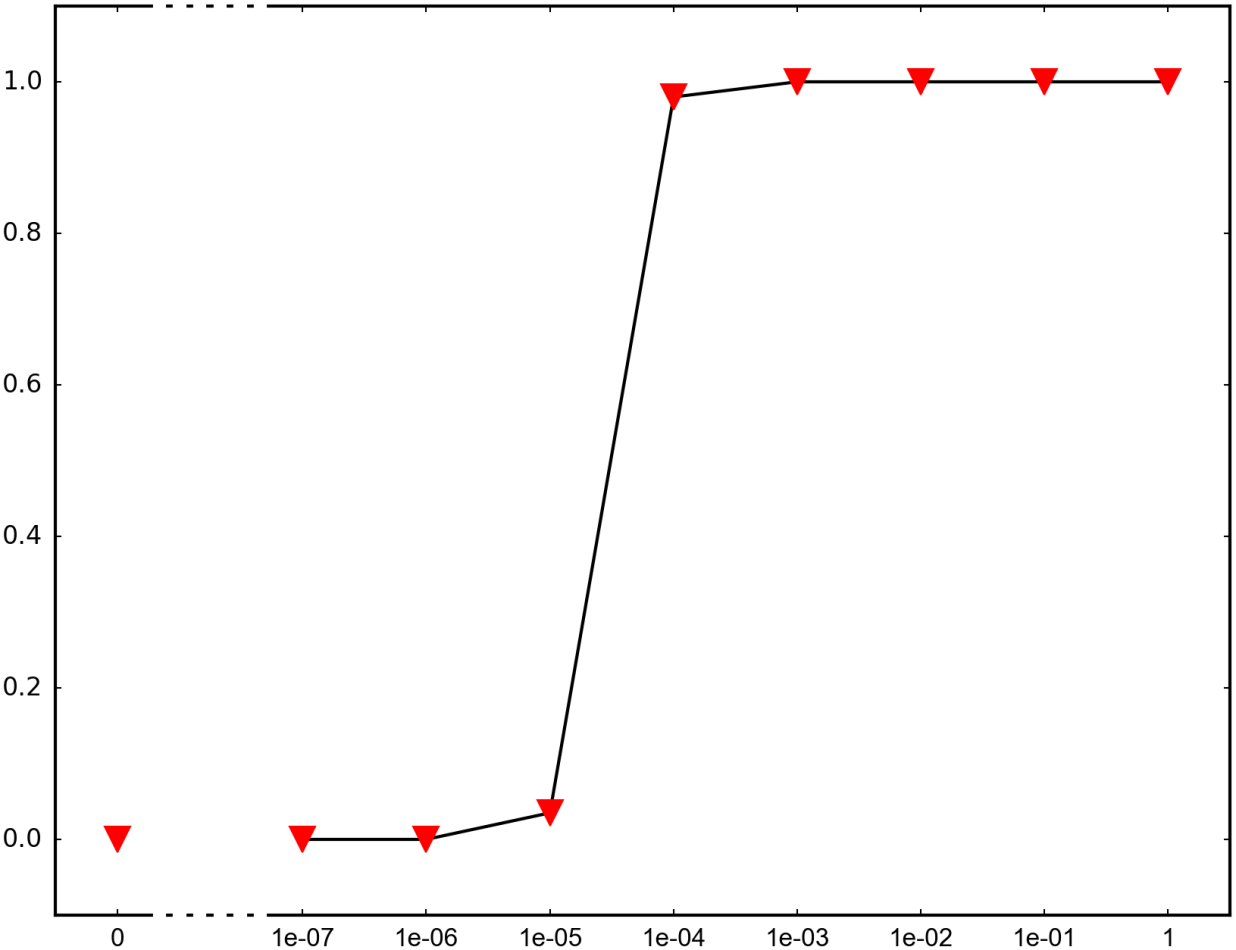
The graph shows the effect of recombination for a 3-locus system with 3 suboptimal peaks. The genotype 111 is the global peak and the genotypes 100, 010 and 001 are suboptimal peaks. Initially, the population is equally distributed on the three suboptimal peaks. The simulation is intended to capture a critical part of an evolutionary scenario for subdivided populations.

**Fig 6:**
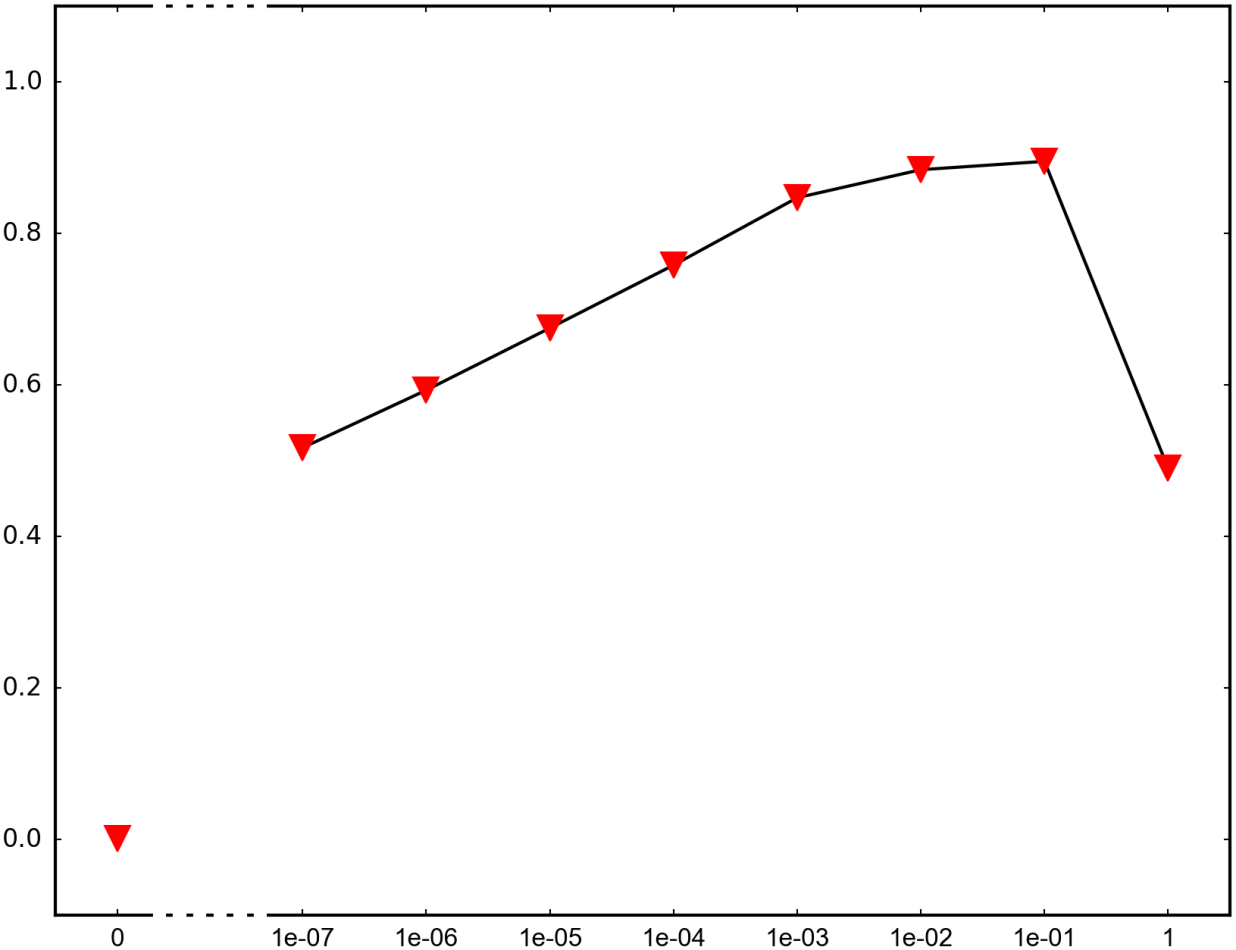
The graph shows the effect of recombination for a 4-locus system with 2 suboptimal peaks. The genotype 1111 is the global peak, and the genotypes 1100 and 0011 are suboptimal peaks. The population is subdivided with migration rate 0.01, and migration takes place every 100th generation. The starting point is a 0000 population. The fitness landscape is the same as in Fig 4.

**Fig 7:**
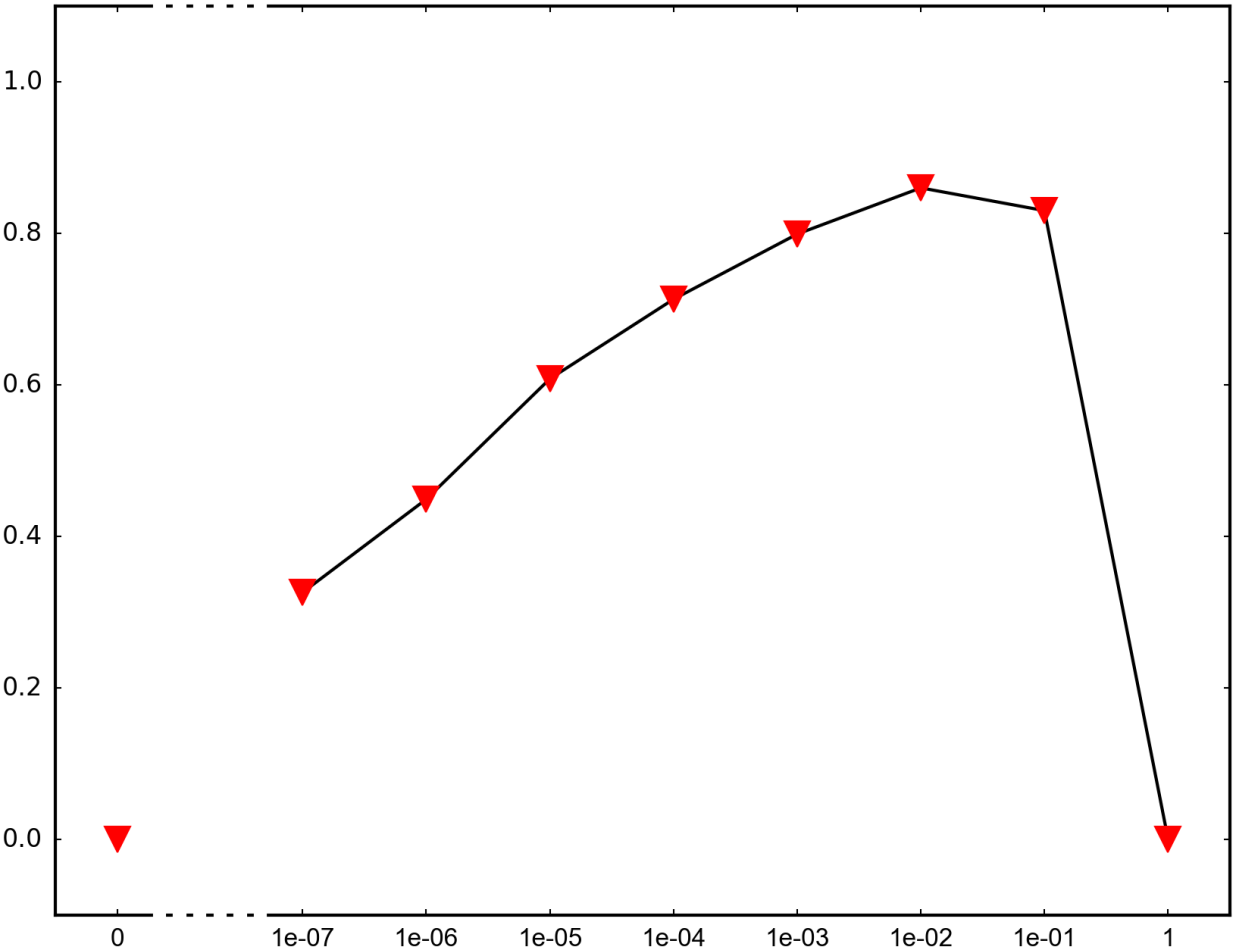
The graph shows a system similar to Fig 6, except that the fitness of the global peak is lower.

**Fig 8:**
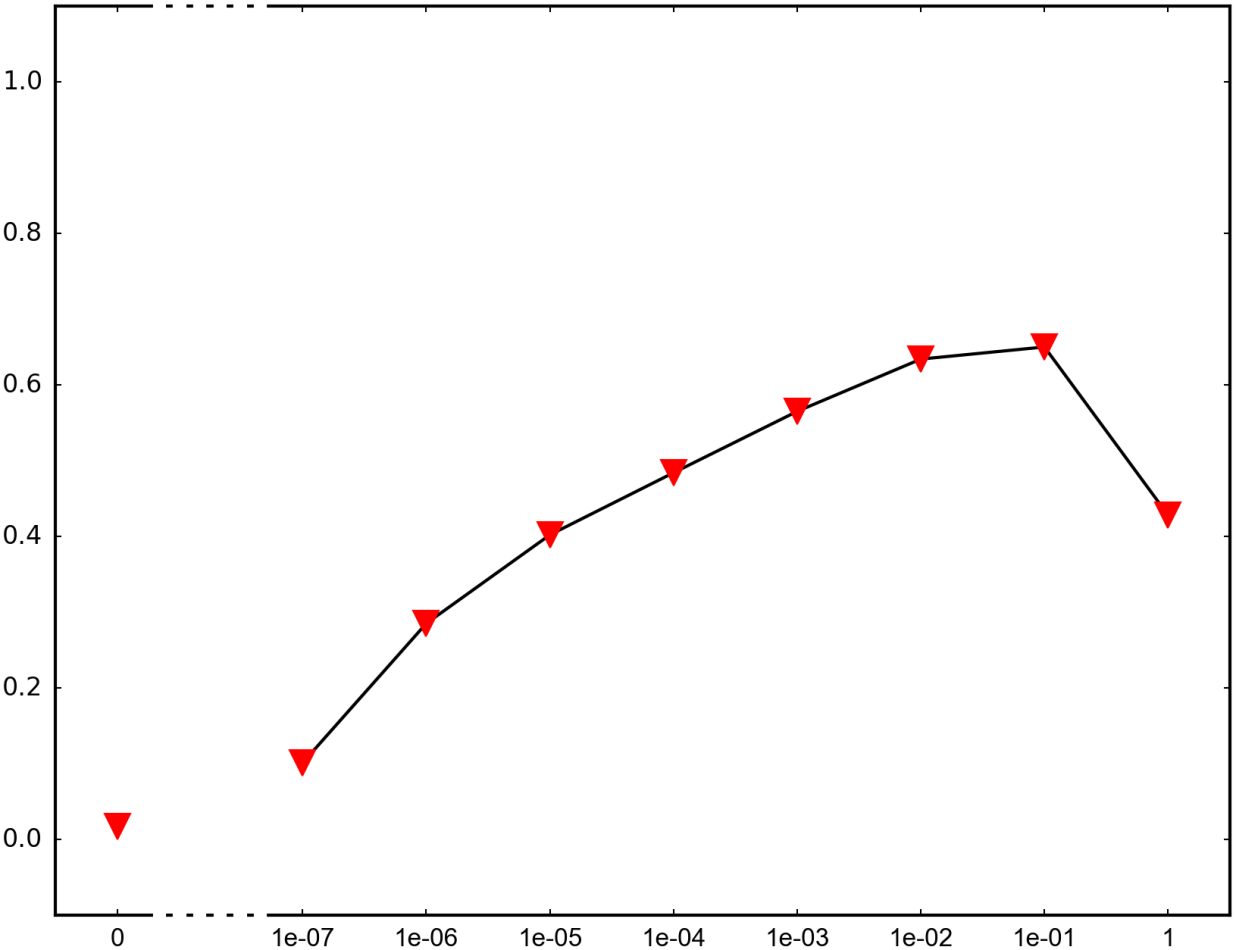
The graph shows a system similar to Fig 6, except that the population size is smaller.

**Fig 9:**
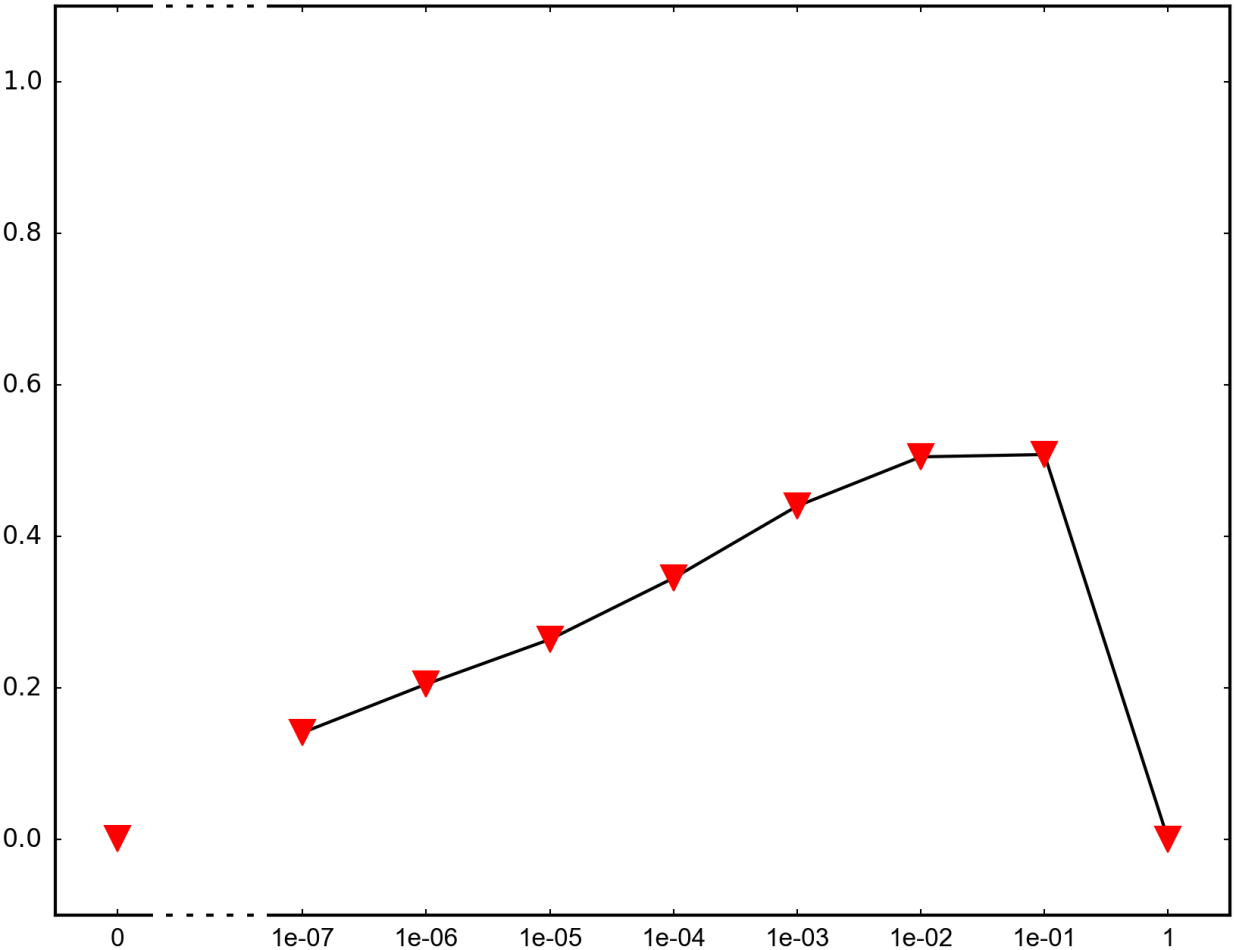
The graph shows the effect of recombination for a 4-locus system with two suboptimal peaks. The genotype 1111 is the global peak, and the genotypes 1100 and 0011 are suboptimal peaks. The population is unstructured and the starting point for adaptation is the wild-type 0000.

**Fig 10:**
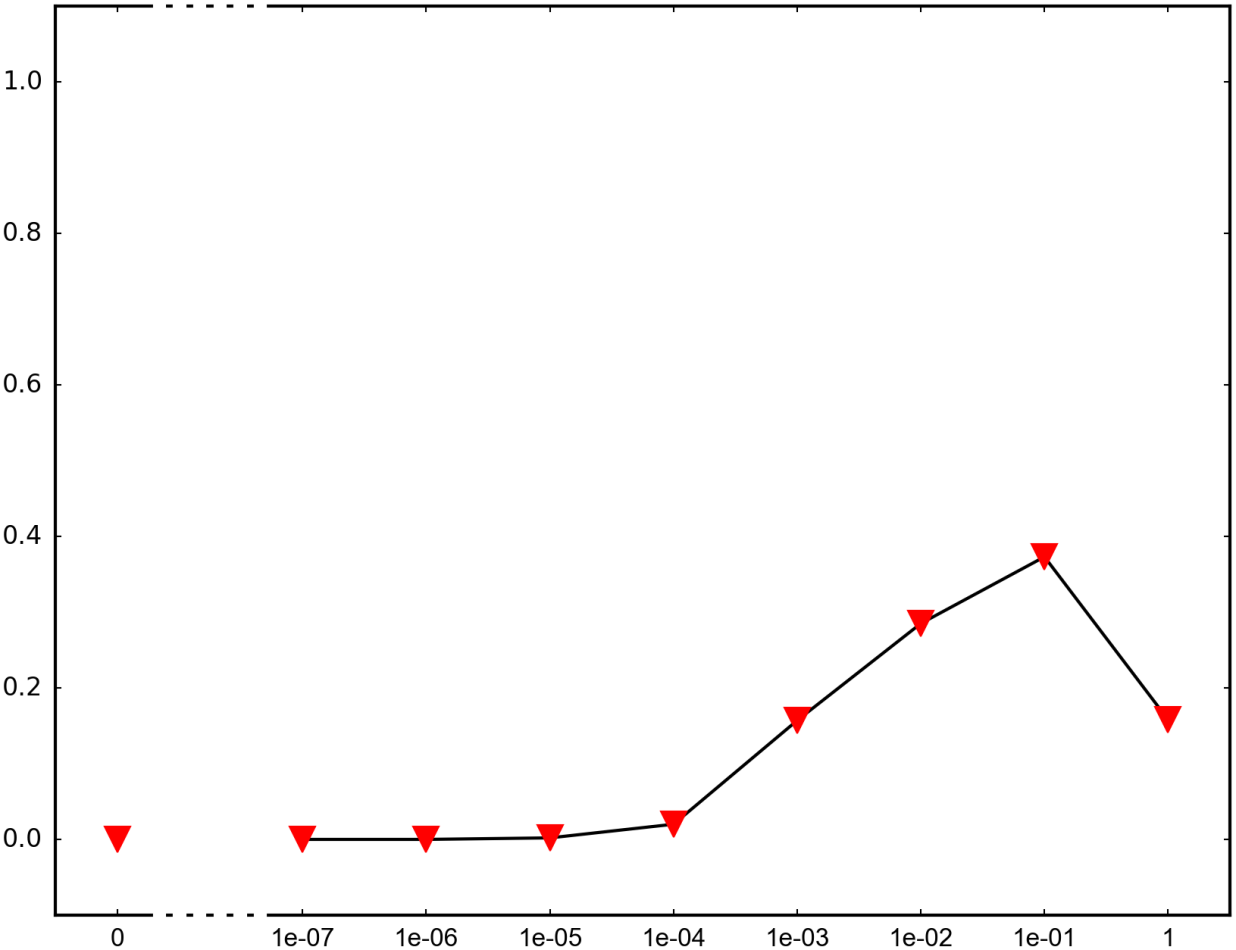
The graph shows the effect of recombination for a 3-locus system with 3 suboptimal peaks. The genotype 111 is the global peak and the genotypes 100, 010 and 001 are suboptimal peaks. The population is unstructured and the starting point for adaptation is the wild-type 000.

**Fig 11:**
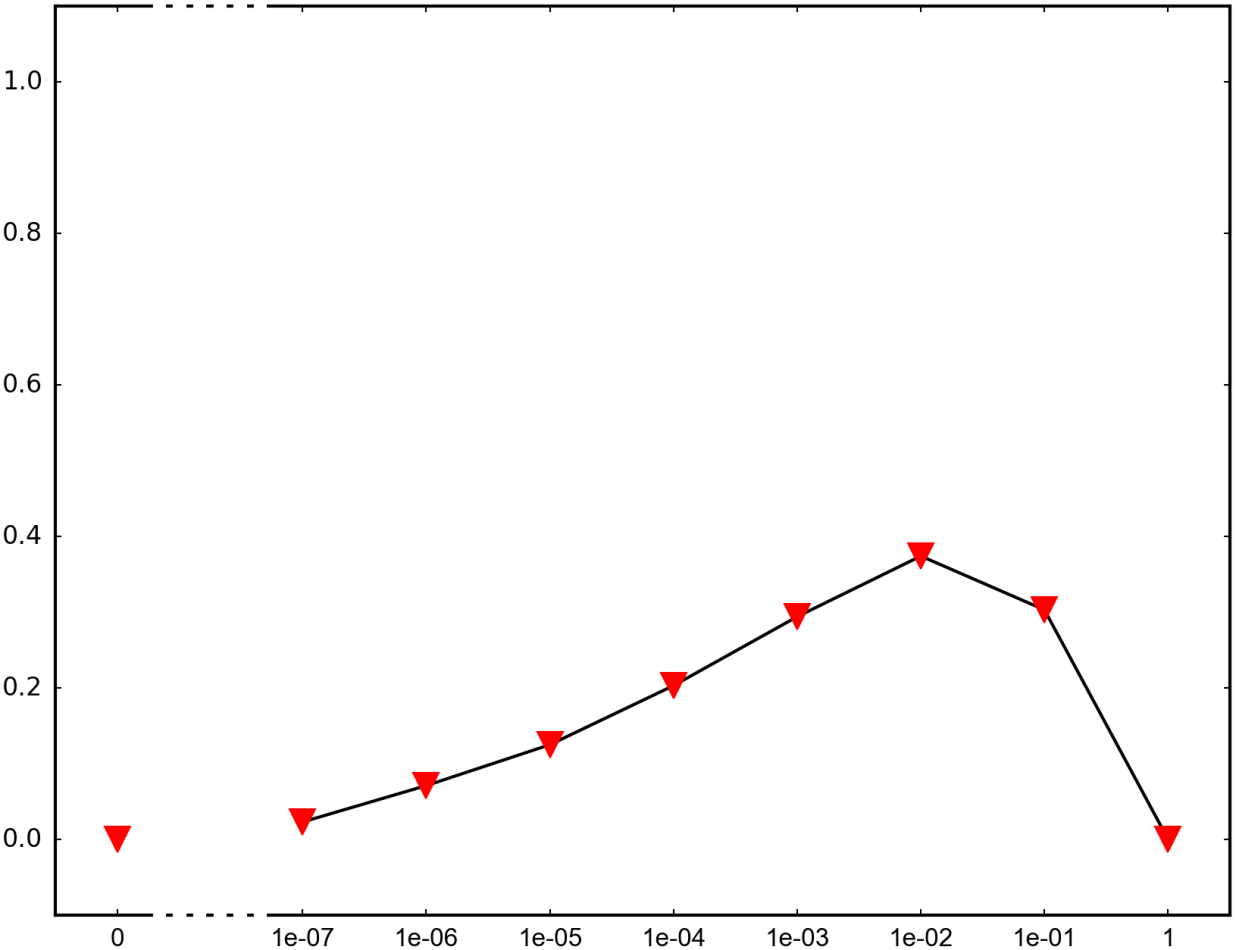
The graph shows the effect of recombination for the 6-locus system with 2 suboptimal peaks. The genotype 111111 is the global peak, and the genotypes 111000 and 000111 are suboptimal peaks. The population is unstructured and the starting point for adaptation is the wild-type 000000.

**Fig 12:**
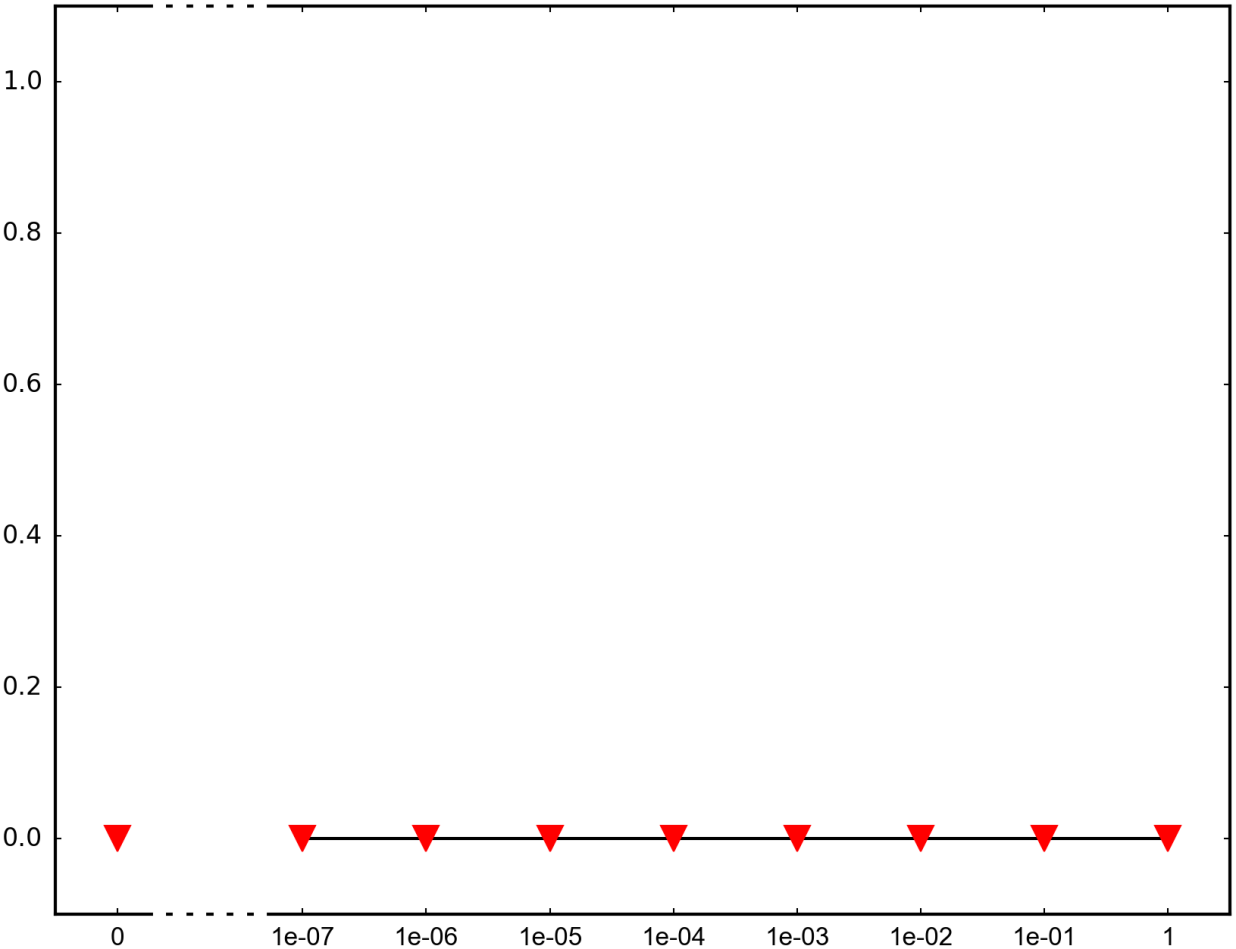
The simulation concerns the effect of recombination for a 2 locus-system with one suboptimal peak. Peak jumping is not possible for this system. The purpose of the simulation is to compare simple valley crossing and peak jumping.

In order to determine the impact of recombination, we repeated each simulation 1000 times and recorded how many times adaptation was successful in the sense that the global peak genotype reached fixation. For each fitness landscape and population structure, we investigated recombination rates between 10^−7^ and 1 (full recombination), as well as 0 (no recombination). The results are summarized in Figs 4–12 where the horizontal axis shows the recombination rate *r* and the vertical axis the estimated probability that the global peak genotype reaches fixation. All parameters, except the recombination rate, are fixed in each figure. The simulation in Fig 12 is intended for comparison between peak jumping and simple valley crossing. The fitness landscape has a suboptimal peak, but peak jumping is not possible. For details on how the simulations were performed, including all parameters, see Methods.

In brief, the simulations in Figs 4–8 concern subdivided population. The starting point for the simulations in Figs 4 and 5 are homogeneous subpopulations consisting of suboptimal peak genotypes. The simulations concern adaptation after the subpopulations have mixed. The simulations in Figs 6–8 are based on a conventional island model with migration. The conditions are most favorable in Fig 6, followed by one simulation with less favorable fitness landscape (Fig 7), and one with smaller than optimal population size (Fig 8). The simulations in Figs 9–11 concern unstructured, that is, well-mixed populations.

The results of the simulations agree well with our theory, as well as intuitive expectations. Adaptation is successful for at least some recombination rates in each setting (Figs 4–11). In contrast, adaptation is almost never successful in the absence of recombination according to the figures. The most striking result is probably that rare recombination (including *r* = 10^−7^) facilitates adaptation for several fitness landscapes. Altogether, the simulations show that recombination is a powerful mechanism for escaping suboptimal peaks.

Notice that frequent recombination is not always a successful strategy. Intuitively, this should make sense since the global peak genotype may combine poorly with mutational neighbors. The optimal recombination rate is strictly between 0 and 1 in several figures. This finding agrees well with Theorem 1.

The simulations indicate that the peak jumping effect is especially important for subdivided populations. Some caution should be exercised when interpreting Figs 4 and 5. The simulations are intended to capture a critical part of an evolutionary scenario for subdivided populations, rather than investigating a complete process. The simulations show that adaptation is successful in “soups of suboptimal peak genotypes” for a wide range of recombination rates. However, the total effect of recombination depends on the entire evolutionary process. The island model with migration (Figs 6–8) can be considered a complement to the simulations in Figs 4 and 5. The results confirm that the peak jumping effect is important in structured populations. Moreover, the effect is robust in the sense that a less favorable fitness landscape (Fig 7), or a smaller than optimal population size (Fig 8), reduces the advantage of recombination only moderately.

The simulations of well-mixed populations (Figs 9–11) demonstrate that the peak jumping effect can be important in unstructured populations as well. The simulations in Figs 6 and 9 differ only in the assumptions on population structure. Recombination is beneficial in both cases, but the effect is clearly stronger for subdivided populations. The conditions are less favorable in Figs 10 and 11, as compared to Fig 9. The fitness landscape has more suboptimal peaks (3 rather than 2) in Fig 10, and the valley between suboptimal peaks is wider in Fig 11. As expected, adaptation is more difficult in Figs 10 and 11 as compared to Fig 9, in the sense that fewer recombination rates facilitate adaptation.

The simulation in Fig 12 is intended for comparisons between simple valley crossing and peak jumping. The depth of the valleys and other factors are similar to the other simulations. The simulations showed no advantage of recombination, regardless of recombination rates.

## Discussion

We have demonstrated an extreme advantage of recombination for a category of complex fitness landscapes. We refer to the mechanism as “peak jumping”, since shuffling of peak genotypes generates a new peak (the move is from peaks to peak, rather than from valleys to peak).

We have shown that sufficiently rare recombination facilitates adaptation for a category of complex fitness landscapes. The peak jumping effect will sometimes require that recombination is relatively infrequent, and we have provided a sufficient condition on the recombination rate *r*. The theoretical condition on *r* does not hold if stochasticity is introduced.

As a complement to the theoretical results, we performed simulations in order to establish thresholds for *r*, and the strength of the effect. As one would expect, the advantage of recombination due to peak jumping depends on both the fitness landscape and population parameters. We verified that the advantage is sensitive for the potential fitness gains because of peak jumping, the number of suboptimal peaks (it is easier to jump from two peaks than from three peaks), the width of the valleys, as well as population size and structure. The simulations confirm that recombination can be advantageous for large, well-mixed populations (of the order 10^7^ individuals), and for relatively small subdivided populations (of the order 10^4^ individuals). Recombination is advantageous for rates as low as 10^−7^ for many landscapes, and usually the optimal rate for adaptation is less than 1. This observation agrees well with our theoretical results.

The peak jumping effect can be dramatic. Indeed, no recombination may lead to failed adaptation, also if one runs the simulation for 50,000 generations (not shown), whereas recombining populations adapt fast. For comparison, we performed a simulation of ordinary valley crossing for a 2-locus system where the depth of the valleys and other factors were similar to peak jumping landscapes. However, adaptation failed regardless of recombination rate.

Our simulations were intended as proof of principle, rather than an extensive study of optimal *r*-values. However, the simulations reveal interesting dynamics and a more detailed study would be valuable. In any case, our simulations show that the peak jumping effect can be very powerful, also for recombination rates realistic for bacteria and viruses.

In the introduction, we briefly discussed different effects of recombination. Peak jumping, the Fisher-Muller effect, and the effect of modularity are somewhat related, and a more thorough comparison is possible at this point. There are clearly similarities in that the benefit of recombination in all three cases depends on allele shuffling generating optimal genotypes. However, what complicates the analysis is that recombination also breaks up good combinations. The former “creative force” dominates over the latter “destructive force” in all cases, but for different reasons.

The Fisher-Muller effect does not assume epistasis. However, a linkage disequilibrium is necessary (double mutants combining beneficial mutations should be underrepresented during the course of adaptation), see e.g. [5, 19] for more background. Importantly, according to most models, the Fisher-Muller effect is fairly small for systems with few loci [5]. Shutting off recombination may imply delayed adaptation. However, there are no dramatic effects comparable to peak jumping, where shutting off recombination can lead to failed adaptation for long periods.

The modularity effect is essentially a very special case of peak jumping. Strictly speaking, the effect is not a special case of this study because of different assumptions on linkage. The global peak can sometimes be generated from chunks of codes from suboptimal peaks, exactly as for peak jumping. However, the critical assumptions are that the genotype is divided into physical modules, that the fitness effects of modules are independent, and that recombination rarely breaks the modules apart. Consequently, recombination can swiftly incorporate favorable modules into the same genome.

Notice that the peak jumping effect differs from the other two effects in that full recombination does not necessarily work well. Even if the global peak genotype can be built from chunks of codes from suboptimal peaks, there is no physical linkage which preserves the favorable chunks of codes. Moreover, peak jumping goes beyond “patching together universally good pieces of code,” in contrast to the other effects. For instance, code from three suboptimal peaks can be combined into a global peak genotype, even if the suboptimal peaks do not combine well pairwise (see Example 1).

One of the classical problems in population genetics is to explain why organisms recombine. Some models suggest that shutting off the mechanism would be advantageous. We suggest that peak jumping can explain maintenance of rare recombination. The argument relies on observing an asymmetry. Sufficiently rare recombination is never a major disadvantage; potential negative effects will shrink by decreasing recombination rate. In contrast, rare recombination can be a major advantage because of peak jumping, in the sense that the population can adapt faster. An organism is likely to undergo several fitness landscapes over time. It seems very possible that occasional peak jumping landscapes could be sufficient for maintaining recombination as a mechanism. However, some caution is necessary. It has been verified that one needs to distinguish between benefits of recombination, such as faster adaptation, and the fate of recombination as a mechanism. Recombination modifiers can be used for modeling the fate of recombination as a mechanism. However such a study is beyond the scope of this paper. For more background on this problem, see e.g. [5] and references therein.

One of the most favorable situations for peak jumping is probably subdivided populations with regular mixing of subpopulations. For subdivided populations, chances are good that all the necessary peak genotypes are available in the global population. The fact that recombination sometimes is especially advantageous for subdivided populations is well established e.g. [5]. For some striking cases, see also [15]. Our observation that rare recombination works better than frequent recombination agrees with other studies of complex fitness landscapes [7, 10, 11]. It is at least theoretically possible that infrequent recombination, as opposed to full recombination, is more likely to facilitate adaptation.

Whether the peak jumping effect is important or not is an empirical question. As mentioned, the evolutionary origin of HIV [9] and species transition for primate lentiviruses, as well as the spread of mosaic HIV-types, can be interpreted as empirical support. The TEM-family of *β*-lactamases provides an intriguing example as well. In general, empirical fitness landscapes are many times complex with multiple peaks e.g. [1–4]. Notice also that there are theoretical arguments in support of that an adapting organism will show more complex gene interactions over time [20, 21].

The category of landscapes we discuss is obviously not large. Indeed, we focused on landscapes where the global peak is difficult to access, but can be generated by shuffling suboptimal peaks. However, related peak jumping scenarios may be as important as the one we have described. For instance, it is plausible that new or altered functions for microbes can develop in special environments only, but are broadly useful in a fully developed form. In such a case, recombination would imply that chunks of codes for different useful functions can be incorporated into the same genome. As in our case, shuffling of suboptimal peaks produces a genotype of higher fitness. The mechanism is exactly the same, except for the fact that different environments are the starting point. The conditions for peak jumping seem especially favorable for microbes because of population size and structure. However, peak jumping could be important for other organisms as well.

A few recent studies analyze the effect of recombination on complex fitness landscapes [7, 10, 11, 22]. The results point in slightly different directions, depending on assumptions and how the problem is phrased. Recombination is sometimes described as a disadvantage. However, we argue that extreme effects of recombination, such as peak jumping, may be more important than how the majority falls, i.e., whether recombination more frequently accelerates or decelerates adaptation.

In a limited sense, our findings lend support to Sewall Wright’s point of view that adaptation may be faster in subdivided populations because of genetic diversity. However, the mechanism in our case relies on suboptimal peaks. The peak jumping effect observed has no correspondence in the two-locus case, or for smooth landscapes. It would be interesting to further explore effects of recombination specific to complex fitness landscapes, especially since recent studies suggest that higher order gene interactions are prevalent in natural populations [23–25].

## Methods

*Models.* We use a Fisher-Wright type model to simulate evolution of finite populations. In brief, for a population consisting of *N* haplotypes the selection step is followed by recombination and then mutation. Recombination is modeled so that for the resulting genotype, each locus is equally likely to agree with either parent’s allele (not taking mutations into account). Moreover, we assume that the alleles are independently sampled for the resulting genotypes, i.e., no linkage is assumed. The mutation rate *μ* refers to the probability for a single mutation per locus and generation. Double (or higher) mutations are allowed.

In mathematical terms, we represent the population as a vector. For example, if *N* = 1000 and the number of individuals with genotype 00, 10 and 01 is 975, 16 and 9, respectively, then the population vector equals (975, 16, 9, 0). From the vector we construct a pool for generating the offspring.

We first describe how the pool is constructed in the case *r* = 1 (full recombination). First selection is performed as multiplication by weights according to the fitness of each genotype. Then recombination and finally mutation are performed as matrix multiplications according to our assumptions. The vector obtained from the population vector after selection, recombination and mutation constitutes the pool for generating offspring. The pool is constructed similarly for *r* = 0 (no recombination), except that there is no recombination step.

For 0 < *r* < 1, the selection step is followed by the recombination and mutation steps for the part of the population that recombines, and just the mutation step for the remaining part of the population.” In mathematical terms, let *v* denote the vector one would obtain after selection and recombination in the case *r* = 1, and *w* the vector after selection in the case *r* = 0. Then the pool vector is obtained as a product of the mutation matrix and the vector *rv* + (1 − *r*)*w*.

Finally, *N* individuals are chosen from the pool by multinomial sampling, according to the proportions given by the pool vector, and the process can start over again. The GNU Scientific Library was used for the multinomial sampling (see below). Notice our model depends on the simplification that the stochasticity is restricted to a single step over a full life cycle. The simplification is reasonable in this setting.

*Code.* All programs were written in C. The Monte Carlo aspects of our simulations used the GNU Scientific Library [26]. The Mersenne Twister [27] was used to generate pseudo-random numbers.

*Specifics on the simulations.* The simulations shown in Figs 4–11 concern peak jumping. The simulation in Fig 12 is intended as comparison. For the simulations in Figs 4, 5, 9 and 12, we ran the simulation for 10,000 generations and recorded how many times the global peak genotype reached fixation; and likewise for simulations in Figs 6, 7, 8, 10 and 11, except that we ran the simulations for 1000 generations. For all simulations, 99 percent was used as the threshold for fixation. The threshold was chosen for computational convenience and does not impact the results. We compared the outcome for different recombination rates *r*, including 0 (no recombination) and 1 (full recombination), and several values in the interval 10^−7^ ≤ *r* ≤ 1. For each value of *r*, the simulation was repeated 1000 times. The results are shown in Figs 4–12. The parameters for each simulation is given below, including the population size *N*, the mutation rate *μ* and the recombination rate *r*, as defined in this section.

**Simulation shown in** Fig 4. *N* = 2 ⋅ 10^7^, *μ* = 10^−9^, and the fitness values are as follows:

$$\begin{array}{c} w_{0000}=1,w_{1000}=w_{0100}=w_{0010}=w_{0001}=1.1,w_{1100}=w_{0011}=1.2,w_{1111}=3,\\ w_{1010}=w_{1001}=w_{0110}=w_{0101}=w_{1110}=w_{1101}=w_{1011}=w_{0111}=0.1. \end{array}$$

The genotype 1111 is the global peak, and the genotypes 1100 and 0011 are suboptimal peaks. The initial population is equally distributed on two suboptimal peaks 1100 and 0011, and the population is well-mixed. The simulation determines the proportion of the population that goes to fixation at the global peak 1111 for each value of the recombination rate *r*.

**Simulation shown in** Fig 5. *N* = 3 ⋅ 10^7^, *μ* = 10^−9^ and the fitness values are as follows:

$$\begin{array}{c} w_{000}=1,\;w_{100}=w_{010}=w_{001}=1.1,\;w_{110}=w_{101}=w_{011}=0.1,\;w_{111}=3. \end{array}$$

The genotype 111 is the global peak, and the genotypes 100, 010, 001 are suboptimal peaks. The initial population is equally distributed on the three suboptimal peaks 100, 010, 001, and the population is well-mixed. The simulation determines the proportion of the population that goes to fixation at the global peak 111 for each value of the recombination rate *r*.

**Simulation shown in** Fig 6. *N* = 2 ⋅ 10^6^, *μ* = 10^−7^ and the fitness values are as follows:

$$\begin{array}{c} w_{0000}=1,w_{1000}=w_{0100}=w_{0010}=w_{0001}=1.1,w_{1100}=w_{0011}=1.2,w_{1111}=3,\\ w_{1010}=w_{1001}=w_{0110}=w_{0101}=w_{1110}=w_{1101}=w_{1011}=w_{0111}=0.1. \end{array}$$

The simulation concerns a 2-island population with migration. The migration rate is 0.01 and migration takes place every 100th generation. The genotype 1111 is a global peak, and the genotypes 1100 and 0011 are suboptimal peaks. All individuals in the initial population have genotype 0000. The simulation determines the proportion of the population that goes to fixation at the global peak 1111 for each value of the recombination rate *r*.

**Simulation shown in** Fig 7. *N* = 2 ⋅ 10^6^, *μ* = 10^−7^ and the fitness values are as follows: The fitness values are as follows:

$$\begin{array}{c} w_{0000}=1,w_{1000}=w_{0100}=w_{0010}=w_{0001}=1.1,w_{1100}=w_{0011}=1.2,w_{1111}=1.3,\\ w_{1010}=w_{1001}=w_{0110}=w_{0101}=w_{1110}=w_{1101}=w_{1011}=w_{0111}=0.1. \end{array}$$

The simulation concerns a 2-island population with migration. The simulation differs from the simulation in Fig 6 only in that the global peak genotype 1111 has lower fitness. The simulation determines the proportion of the population that goes to fixation at the global peak 1111 for each value of the recombination rate *r*.

**Simulation shown in** Fig 8. *N* = 2 ⋅ 10^4^, *μ* = 10^−5^, and the fitness values are as follows:

$$\begin{array}{c} w_{0000}=1,w_{1000}=w_{0100}=w_{0010}=w_{0001}=1.1,w_{1100}=w_{0011}=1.2,w_{1111}=3,\\ w_{1010}=w_{1001}=w_{0110}=w_{0101}=w_{1110}=w_{1101}=w_{1011}=w_{0111}=0.1. \end{array}$$

The simulation concerns a 2-island population with migration. The simulation differs from the simulation in Fig 6 only in that the population is smaller and the mutation rate is lower. The simulation determines the proportion of the population that goes to fixation at the global peak 1111 for each value of the recombination rate *r*.

**Simulation shown in** Fig 9. *N* = 10^6^, *μ* = 10^−7^ and the fitness values are as follows

$$\begin{array}{c} w_{0000}=1,w_{1000}=w_{0100}=w_{0010}=w_{0001}=1.1,\;w_{1100}=w_{0011}=1.2,\;w_{1111}=3,\\ w_{1010}=w_{1001}=w_{0110}=w_{0101}=w_{1110}=w_{1101}=w_{1011}=w_{0111}=0.1. \end{array}$$

The genotype 1111 is the global peak, and 1100 and 0011 are suboptimal peaks. All individuals in the initial population have genotype 0000, and the population is well-mixed. The simulation determines the proportion of the population that goes to fixation at the global peak 1111 for each value of the recombination rate *r*.

**Simulation shown in** Fig 10. *N* = 10^6^, *μ* = 10^−7^, and the fitness values are as follows:

$$\begin{array}{c} w_{000}=1\;w_{100}=w_{010}=w_{001}=1.1,\;w_{110}=w_{101},w_{011}=0.1,\;w_{111}=3. \end{array}$$

The genotype 111 is the global peak, and the genotypes 100, 010 and 001 are suboptimal peaks. All individuals in the initial population have genotype 000, and the population is well mixed. The simulation determines the proportion of the population that goes to fixation at the global peak 111 for each value of *r*.

**Simulation shown in** Fig 11. *N* = 10^7^, *μ* = 10^−8^ and the fitness values are as follows:

$$\begin{array}{c} w_{000000}=1,\;w_{100000}=w_{010000}=w_{001000}=w_{000100}=w_{000010}=w_{000001}=1.1,\\ w_{110000}=w_{101000}=w_{011000}=w_{01100}=w_{000110}=w_{000101}=w_{000011}=1.2,\\ w_{111000}=w_{000111}=1.3\;\text{and}\;w_{111111}=3. \end{array}$$

The remaining genotypes have fitness 0.1. The genotype 111111 is the global peak, and the genotypes 111000 and 000111 are suboptimal peaks. All individuals in the initial population have genotype 000000, and the population is well mixed. The simulation determines the proportion of the population that goes to fixation at the global peak 111111 for each value of the recombination rate *r*.

**Simulation shown in** Fig 12. *N* = 10^7^, *μ* = 10^−8^, and the fitness values are as follows:

$$\begin{array}{c} w_{00}=1,w_{10}=0.1,w_{11}=0.1,w_{11}=3. \end{array}$$

The genotype 11 is the global peak and 00 is a suboptimal peak. Peak jumping is not possible for this system. The purpose of the simulation is to compare simple valley crossing and peak jumping. All individuals in the initial population have genotype 00, and the population is well-mixed. The simulation determines the proportion of the population that goes to fixation at the global peak 11 for each value of the recombination rate *r*. It should be noted that recombination can facilitate simple valley crossing under different assumptions, e.g. [6].
